# Effects on individual level behaviour in mackerel (*Scomber scombrus*) of sub-lethal capture related stressors: Crowding and hypoxia

**DOI:** 10.1371/journal.pone.0213709

**Published:** 2019-03-13

**Authors:** Neil Anders, Kirsten Howarth, Bjørn Totland, Nils Olav Handegard, Maria Tenningen, Michael Breen

**Affiliations:** 1 Fish Capture Division, Institute of Marine Research (IMR), Bergen, Norway; 2 Department of Biological Sciences, University of Bergen, Bergen, Norway; 3 Marine Ecosystem Acoustics, Institute of Marine Research (IMR), Bergen, Norway; Universidad de Cádiz, Facultad de Ciencias del Mar y Ambientales, SPAIN

## Abstract

Stress to fish during harvest in wild capture fisheries is known to negatively influence subsequent survival in catches that are released. Therefore, if fisheries are to be conducted sustainably, there is a need to promote good fish welfare during the capture process. Purse seine fishing is a widespread and efficient fishing method. However, capture and release of fish from purse seines (a process called “slipping”) can result in extremely high mortality in small pelagic schooling species. The objective of this study was to establish behavioural indicators of sub-lethal stress in Atlantic mackerel (*Scomber scombrus)* that may be used to set safe threshold limits for use in commercial purse seine fishing, in order to ensure good fish welfare and thereby minimise slipping mortality. Controlled mesocosm scale experiments with schools of mackerel in net pens were undertaken to determine behavioural responses to simulated purse seine capture stressors of “crowding”, “hypoxia” and “crowding & hypoxia”. Crowding (at 30 kg.m^-3^) was achieved by reducing the volume of the net pen, while hypoxia (to 40% oxygen saturation) was achieved by surrounding the net pen with a tarpaulin bag to prevent water exchange. Using video analysis, we investigated behavioural responses in nearest neighbour distances, nearest neighbour angular deviations, tail beat amplitude and tail beat frequency (TBF). Of the metrics considered, only TBF showed a response; a significant increase to “crowding” (42% increase) and “crowding & hypoxia” (38% increase) was found. The increase in TBF in response to “hypoxia” alone (29% increase) was not significant. We therefore conclude that increases in tail beat frequency may be used as an indicator of sub-lethal purse seine capture stress in mackerel that may have utility in minimising post slipping mortality.

## Introduction

Stress prior to the escape, release or slaughter of wild fish caught by fishing gear has been shown to reduce subsequent fitness [1], survival [2–4] and resulting product quality [5–9]. Therefore, the minimisation of stress to promote good fish welfare during capture should be of paramount concern for ethical, sustainable and profitable wild capture fisheries.

In order to promote good welfare, objective and easily measurable indicators of stress are required. Physiological measures may be used [10] and have been widely applied in studies examining stress responses to fisheries capture [7,11,12]. However, physiological responses can be difficult to collect in the field, are easily confounded by the experimenter [10] and can lack concordance with mortality [13,14]. Behavioural indicators of stress are, however, relatively easy to observe, non-invasive to record and potentially non-intrusive [15]. They are also a more holistic and often more immediate expression of the stress response, reflecting underlying neurological and physiological changes [16–19].

Purse seine fishing targets aggregations of pelagic schooling species, by surrounding the school with a net to trap them. As the net volume is reduced by hauling the net aboard, catches are concentrated to a sufficient degree to allow effective pumping or brailing onboard the vessel. Escape of fish through the net is not usually feasible due to the small mesh size used in most sections of the purse seine. Capture by purse seine accounts for up to 30% of global fisheries capture [20], but knowledge regarding fish welfare in response to this capture method is currently limited [21]. However, it has been shown that the confinement, subsequent crowding and associated hypoxia [12] as the net volume is reduced is highly stressful for the captured fish and can result in high mortality rates if unwanted catches are released [12,22–24]. The process of releasing unwanted catches from purse seines whilst still in the water is termed “slipping”. The reasons for slipping vary [25] but the practice is widely employed in a variety of different purse seine fisheries [12,23,26,27]. If high mortality rates in slipped catches are to be avoided, there is a need to develop reliable indicators of stress in purse seine fishing so that impacts upon fish welfare during the capture process can be minimised and subsequent survival promoted.

A fundamental life history strategy for obligatory schooling pelagic species is the formation of shoals or schools with conspecifics, and highly complex behavioural responses to stress can be observed at this collective level [28–31]. However, school structure and cohesion are maintained by adherence of individual fish to simple behavioural rules, consisting of far field attraction and near field repulsion, from which neighbour directional alignment arises [32]. Resulting collective schooling behaviour and its associated fitness advantages [28,33–36] therefore arise due to the summation of many individual behavioural interactions [37,38]. Stressors are known to influence the behavioural rules governing schooling by altering spacing [39] and polarisation between individuals [40]. In turn, individual swimming speed may change in response to stress [41,42], and further modify the attraction/repulsion and alignment rules [43]. Individual behavioural change therefore has the potential to help explain school level collective responses, and is an important component for understanding the behavioural stress response in schooling species.

Quantification of the three-dimensional schooling behaviour of individual fish can be challenging [44]. Previous work has tended to focus on using photogrammetry [45,46,47] or stereophotogrammetry [48,49] techniques, sometimes in conjunction with tracking and computer vision [42,50–52]. Using such methods allows the quantification of important schooling parameters such as the degree of cohesion and alignment between individuals [53]. Photogrammetric techniques have also been used to determine activity levels in schooling fish, usually measured as relative swimming speed [45,46,50,52,54] but sometimes in absolute terms [47,55,56]. However, activity levels of fish may also be determined by examination of tail beat frequency (TBF), which may be more informative in determining the welfare status of individuals due to TBF being the main behavioural determinant of swimming speed [57] and its strong correlation with energy expenditure [58,59,60,61]. Consequently, measures of tail beat activity have been successfully used to determine sub-lethal effects and recovery in stressed fish [62,63,64].

In this study, we report on mesocosm scale experiments to determine individual level behavioural stress indicators in response to simulated sub-lethal purse seine capture stressors (hypoxia and crowding) in schools of Atlantic mackerel (*Scomber scombrus*). Mackerel supports extensive (total landings in 2016 in excess of 1 million tonnes [65]) and valuable (2017 catch value in Norway of around 2.1 billion Norwegian krone [66]) fisheries, including those using purse seine (>600 purse seine vessels target mackerel [65]). However, mackerel is a delicate species and is therefore highly susceptible to stress factors during capture and slipping [22,23].

The work described here is part of the same series of experiments as described by Handegard *et al*. [31] in which collective level behavioural responses to stressors were described. However, this study offers a different observational perspective by examining individual fish responses rather than collective behaviours. The objective was to establish quantifiable behavioural indicators of sub-lethal capture stress that may be used to set safe threshold limits for use in commercial purse seine fishing, in order to ensure good fish welfare and thereby minimise slipping mortality.

## Materials and methods

### Fish capture, welfare and husbandry

Adult wild mackerel were caught passively and maintained in aquaculture net pens at the Austevoll Research Station (60°N) of the Institute of Marine Research, Norway, during autumn 2014 and summer and autumn 2016. Fish were voluntarily attracted into a holding net pen (12 x 12 x 12m) at night using small sized aquaculture feed pellets. Here fish were retained and fed daily using pellets, as well as being able to forage naturally on any food items passing into the net pens. Fish caught in 2014 were maintained for approximately 12 months prior to experimentation, and for approximately 4 months if caught in 2016. Mean mackerel (± SD) length, weight and Fulton’s condition factor (K, [67]) was 40.5 ± 2.5cm, 887 ± 161g and 13.35 ± 1.4K respectively.

All experiments were approved by the Norwegian Food Safety Authority. To determine sub-lethal levels of crowding and hypoxic stress and to ensure welfare, a laboratory pilot experiment (FOTS 7601, consisting of replicate groups of n = ~30, detailed fully in [31]) was first conducted to determine levels at which significant increases in mortality were induced. Crowding to 30 kg.m^-3^ and 40% oxygen saturation were found to be safe threshold levels. There was no data regarding anticipated behavioural responses on which to base a power analysis to determine minimal group sizes for the main experiment (FOTS 7671). Therefore, the number of fish used in each replicate was chosen on the basis of anticipated oxygen consumption rates in order to produce hypoxic conditions and to ensure normal schooling and behavioural enrichment in the mesocosm scale experimental set up.

Seven days prior to the start of experimentation, approximately equal numbers of fish (estimated visually) were transferred by voluntarily attracting them into one of four experimental net pens (5 x 5 x 5m) using feed pellets. The maintenance of schooling behaviour and normal feeding activities indicated that the fish acclimatised well to the experimental pens before the experiments began. Fish welfare was monitored daily, with respect to signs of distress and/or injury, prior to and during the experiments. Where necessary, distressed fish were immediately removed and euthanised with an overdose of MS-222 at 500mg/L concentration or by percussive stunning. Pellet feeding was halted 2 days prior to the start of experimental treatment and resumed 1 day after treatment had finished. Further details of fish capture, welfare and husbandry are reported in [31].

### Experimental treatments

**Fig 1:**
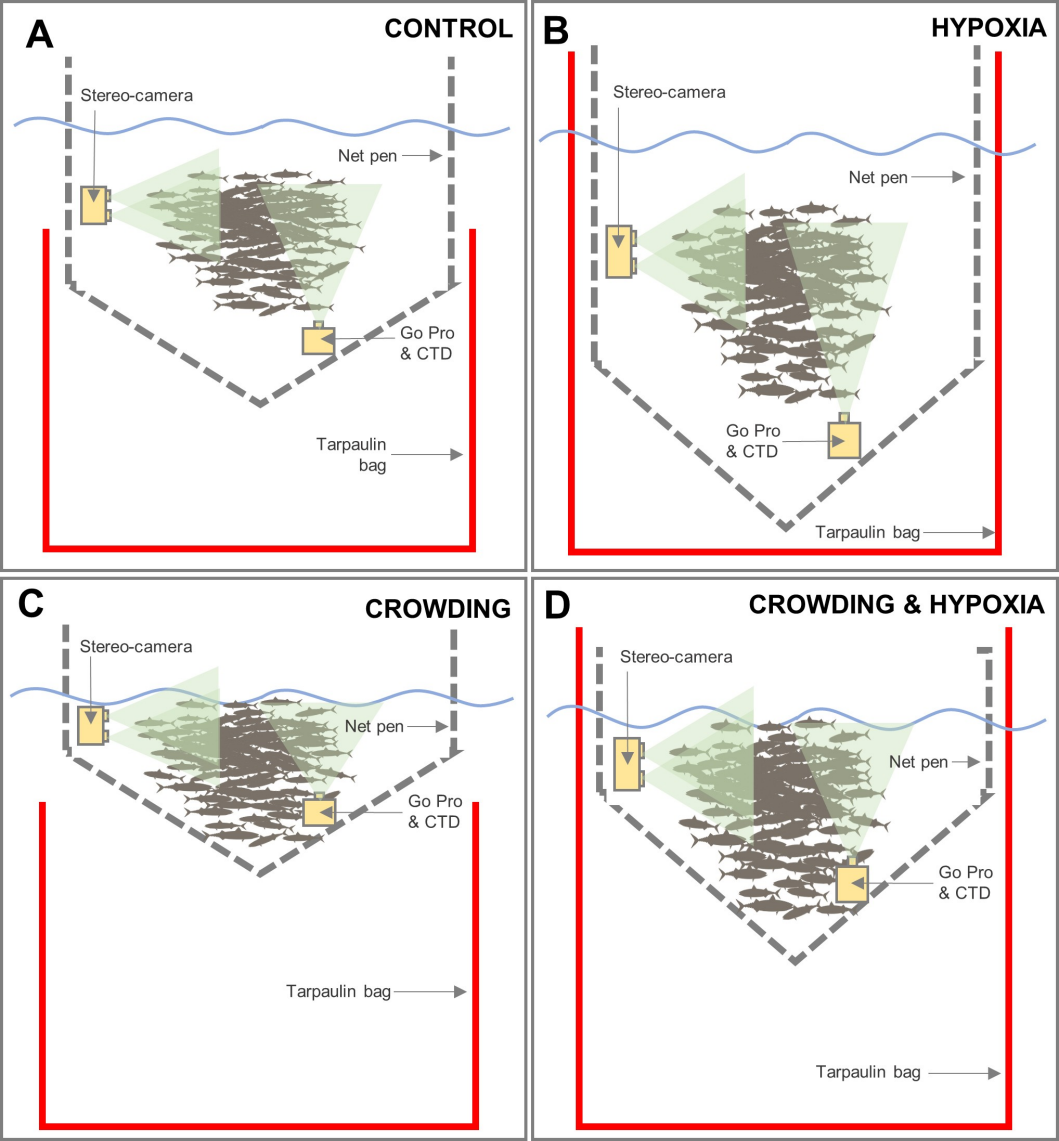
Schematic of the experimental set up. Showing a vertical cross-section of net pens (not to scale) for the different stressor treatments of: **A**: “control”; **B**: “hypoxia”; **C**: “crowding” and **D**: “crowding & hypoxia”. The waters surface is indicated by the wavy blue line. Shaded green areas represent the approximate viewing direction of the cameras.

Only four experimental net pens were available at any one time. The experiment was therefore conducted over three phases (i.e. replicates); one phase in September 2015 and two more phases in September and October 2016 respectively (Table 1). During each phase, separate groups of fish (i.e. each net pen) were each exposed to one of four stressor treatments: either “hypoxia”, “crowding”, “crowding and hypoxia” or a “control” (Fig 1). Hypoxia treatments (Fig 1B & 1D) were achieved by lifting the opening of a surrounding waterproof tarpaulin bag above the water’s surface by hand to prevent the flow of oxygenated seawater into the pen. The respiratory action of the fish then reduced the oxygen saturation to the desired level (40%) over time. Oxygen was monitored by a calibrated RINKO III electronic oxygen sensor (JFE Advantach Co., Ltd.), attached to a SAIV CTD (Model: SD204) recording at 2s intervals with a ±2% accuracy (at 25°C). For non-hypoxic treatments (Fig 1A & 1C), the tarpaulin bag remained in place but with the opening below the surface to allow inflow of oxygenated water. Crowding treatments (Fig 1C & 1D) were achieved by lifting the net pen vertically in the water by hand to reduce the available volume for the fish, until crowded to ~30 kg.m^-3^. For non-crowding treatments (Fig 1A & 1B), the net pen volume was not altered.

**Table 1:** Behaviour monitoring timings. Start time of monitoring of behavioural responses to stressor treatments for each experimental phase. 10 minutes of behavioural video footage was collected during each monitoring period.

			Monitoring period start time (hh:mm after start of treatment)			
Phase	Monitoring period code^1^	Monitoring period description	Control	Crowding	Hypoxia	Crowding & hypoxia
September 2015	P	Pre-treatment	-01:25	-02:36	NA^2^	-01:28
	T1	Start of treatment	00:00	00:00	NA^2^	00:00
	T2	During treatment A	01:31	01:06	NA^2^	01:29
	T3	During treatment B	02:35	02:01	NA^2^	02:31
	M4	1 day post treatment	22:40	26:17	NA^2^	28:31
	M5	2 days post treatment	48:18	44:36	NA^2^	46:46
	M6	3 days post treatment	70:16	70:22	NA^2^	70:27
	M8	6 days post treatment	142:14	143:35	NA^2^	141:51
September 2016	P	Pre-treatment	-01:00	-01:17	-01:00	-01:20
	T1	Start of treatment	00:00	00:00	00:00	00:00
	T2	During treatment A	01:15	01:05	01:16	01:15
	T3	During treatment B	01:45	01:40	01:45	NA^3^
	M1	~0.5 hours after end of treatment	02:40	02:40	02:44	02:05
	M2	~2 hours after end of treatment	04:10	04:10	04:05	03:35
	M3	~4 hours after end of treatment	06:30	06:18	06:05	05:50
	M4	1 day post treatment	28:40	29:45	28:50	26:55
	M5	2 days post treatment	50:18	48:50	48:30	46:55
	M6	3 days post treatment	71:20	73:55	74:30	70:50
	M7	4 days post treatment	95:55	96:43	94:05	95:18
	M8	5 days post treatment	117:59	118:15	118:00	117:59
October 2016	P	Pre-treatment	-01:00	-01:00	-02:15	-00:55
	T1	Start of treatment	00:00	00:00	0:00	00:00
	T2	During treatment A	01:00	01:00	01:40	00:15
	T3	During treatment B	02:00	02:00	02:35	00:31
	M1	~0.5 hours after end of treatment	03:52	02:50	03:25	01:15
	M2	~2 hours after end of treatment	05:27	04:15	04:55	02:45
	M3	~4 hours after end of treatment	07:02	06:14	06:55	04:45
	M4	1 days post treatment	24:45	27:30	27:50	23:40
	M5	2 days post treatment	50:00	48:20	48:22	51:15
	M6	3 days post treatment	75:50	74:30	71:35	75:55
	M7	4 days post treatment	96:10	99:05	96:25	98:35
	M8	5 days post treatment	119:30	121:25	120:40	120:30

^1^Monitoring period codes consist of either; “P” for pre-treatment; “T” for during treatment or “M” for post treatment monitoring

^2^Hypoxia treatment was not applied during the September 2015 phase

^3^There was insufficient time to collect a third monitoring period (T3) during treatment before oxygen levels fell below acceptable welfare levels

Further detail of net pen instrumentation can be found in [31], including a vertically orientated echo-sounder (Simrad EK60 with a Simrad ES120-7C 120kHz transducer) used to provide estimates of fish abundance and density.

### Monitoring behavioural responses

Behavioural responses to treatment were monitored once prior to the application of the stressor and three times during treatment (Table 1). Each monitoring period lasted 10 minutes. Monitoring periods during treatments involving hypoxia corresponded approximately to the times when oxygen levels inside the net pen reached 70%, 50% and 40% saturation. Monitoring periods during the “crowding” and “control” treatments were conducted at the start of treatment, at ~1 hour after the start of treatment and also at ~2 hours after the start of treatment (Table 1).

Behaviour was also monitored for up to 6 days post treatment (Table 1). In the September 2015 phase, observations were collected on one, two, three and six days post treatment. However, to achieve better time resolution, post treatment monitoring was increased in the September and October 2016 phases, to include observations immediately post treatment (0.5, 1 and 2 hours post the end of treatment) and then once daily after that for up to five days (Table 1).

Behavioural responses during monitoring periods were collected using two camera systems, a vertically orientated GoPro HERO 3 or 4, filming in high resolution colour at 1080p at 25fps and a horizontally orientated stereo-camera (Fig 1). The GoPro was attached to the CTD and positioned close to the bottom and off centre of the net pen (Fig 1) using a rope and pulley system, to allow observation of behaviour from below and to avoid the vacuole in the centre of the typically circular schooling fish. Detail of the stereo-camera system setup can be found in the supporting information (S1 Methods). Examples of the footage collected by the GoPro camera are shown in S1 Video.

### Quantifying behavioural responses

Previous work has demonstrated alterations in fish swimming activity in response to hypoxia (eg. [42,68]) and crowding stress (eg. [69–71]), and [41] and [72] demonstrated alterations in swimming speed in response to stress in small pelagic species. We therefore hypothesised that individual mackerel would exhibit changes in swimming speed in response to the stressors. Consequently, we used the footage collected by the vertically orientated GoPro camera to quantify tail beat frequency (TBF) and tail beat amplitude (TBA), both of which influence overall swimming speed. Increases in TBF scale linearly with speed, while TBA tends to stabilise above a critical point with variability only seen at lower speeds [57].

For this, three randomly selected five second clips from within each monitoring period were selected. From within each five second clip, five fish (selected using random coordinates of an overlaid grid applied using Image J software [73]) were selected. TBF (in tail beats per second) in the selected fish was determined for over the whole duration of the appearance of the fish within the field of view of the camera, providing that it was on camera for ≥ 1 second. If not, another random fish was selected. We defined one tail beat as the movement from one extreme lateral position to the opposite position. Mean tail beat amplitude (TBA) was collected using the same fish as TBF, and full detail of the quantification of this metric is included in the supporting information (S2 Methods).

Reducing the available swimming volume of a school of fish by crowding them should affect spacing between individuals and hypoxia has been shown to alter inter-fish distances [39]. We therefore used the stereo-camera footage to determine behavioural responses in nearest neighbour distances (NND) to the treatments. We were also interested in determining how the stressors influenced alignment between individuals [40]. For this, we examined nearest neighbour angular deviation in pitch (ADP) and yaw (ADY) using the stereo-camera footage. Detail of the quantification of these behavioural metrics are included in the supporting information (S2 Methods).

### Confirming sub-lethal stress

Net pens were monitored daily for mortalities. Mortality rate over the whole experiment was 0.36% (51 dead out of ~14000 mackerel used), indicating the levels of stressor treatments we applied were essentially sub-lethal. Full details of mortality monitoring and results are reported in [31].

### Statistical analysis

Exploration of the datasets followed procedures described by [74]. The data was nested, in that multiple observations of the behavioural metric were collected from different video clips, monitoring periods, stressor treatments, net pens and experimental phases. As such, observations within levels were likely to be more similar to one another than to observations between levels, violating the assumption of independence. To avoid the risk of Type 1 errors, mixed modelling techniques were applied following procedures described by [75].

All statistical analysis was undertaken using R version 3.4.2 [76], with the level of significance set at 0.05. TBF (S1 Dataset) represented normally (continuous) distributed data and was modelled using linear mixed models (LMM) using the lme function from the nlme library of R [77]. We first considered only data from the during treatment monitoring periods (ie. “T1”, “T2” and “T3”, Table 1). The correlation structure of “net pen” (up to 11 levels) nested within “monitoring period” (up to 12 levels) nested within “video footage” (the five second clip from the GoPro footage) were incorporated as random effects. “Experimental phase” (up to three levels, September 2015, September 2016 or October 2016) was included as a fixed rather than a random effect due to a lack of levels (minimum required levels for inclusion as a random effect is 6 [78]). The other fixed effects were the treatment conditions: “crowded”, “hypoxia” (dummy variables coded as 1 for presence and 0 for absence) and their interaction. We attempted an interaction between the stressor variables and “experimental phase” but the model would not converge due to missing hypoxia data in the September 2015 phase. We therefore modelled “experimental phase” as an additive effect. Further parameter selection and model reduction was not undertaken. Model assumptions were checked visually using residual plots. The R syntax for the “treatment periods” TBF model was as follows:

(1) lme(TBF ~ Crowded * Hypoxia + Experimental_phase,
  random = ~ 1 | Net_pen/Monitoring_period/Video_footage,
  method = "REML")

We also modelled TBF using data from all monitoring periods. For this, we included fixed effects of an interaction between “monitoring period” (categorical with up to 12 levels, see Table 1 for an explanation of levels) and “crowded” and “hypoxia”. As validation plots indicated differences in residual spread between monitoring periods, we incorporated the heterogeneity into the model using a VarIdent variance structure [75]. Consequently, the R syntax for the “all periods” TBF model was as follows:

(2) lme(TBF ~ Monitoring_period * Crowded * Hypoxia + Experimental_phase,
  random = ~ 1 | Net_pen/Monitoring_period/Video_footage,
  weights = varIdent(form = ~1 | Monitoring_code),
  method = "REML")

The significance of terms in models was determined using likelihood ratio testing. We investigated marginal (variance explained by the fixed effects) and conditional effects (variance explained by the fixed and random effects together) of LMM using the pseudo R^2^ equation of [79].

Detail of the statistical analysis undertaken for the behavioural metrics of TBA (S2 Dataset), NND (S3 Dataset), ADP (S4 Dataset) and ADY (S5 Dataset) is included as supporting information (S3 Methods).

## Results

### Application of the stressor treatments

The numbers of fish within each net pen during each experimental phase (i.e. replicate) were approximately equal, although the September 2015 phase had considerably lower abundance (Table 2). Mean numbers of fish in each net pen for the September 2015, September 2016 and September 2016 were 586, 1314 and 1645.

**Table 2:** Experimental design. Numbers of fish exposed to stressor treatments and associated rates of oxygen decline throughout the three phases of the experiment.

Phase	Stressor treatment	Treatment date	Number of fish	Oxygen minimum during treatment (% saturation)	Oxygen minimum during treatment (mg/L)	Rate of oxygen decline during treatment (%/hour)	Rate of oxygen decline during treatment (%/fish/hour)
September 2015	Crowded	10/09/2015	669	96.81	7.84	NA	NA
	Crowded & hypoxia	09/09/2015	503	36.86	2.98	11.4	0.023
	Control	11/09/2015	*	103.39	8.39	NA	NA
September 2016	Crowded	28/09/2016	951	87.78	6.88	NA	NA
	Hypoxia	27/09/2016	1282	36.35	2.83	16.8	0.013
	Crowded & hypoxia	30/09/2016	1185	36.90	2.97	26.4	0.022
	Control	29/09/2016	1838	91.58	7.23	NA	NA
October 2016	Crowded	26/10/2016	1662	86.82	7.08	NA	NA
	Hypoxia	25/10/2016	1230	38.30	3.33	8.4	0.007
	Crowded & hypoxia	27/10/2016	1795	38.69	3.30	39	0.021
	Control	24/10/2016	1891	85.49	7.37	NA	NA

* = missing echosounder data

**Fig 2:**
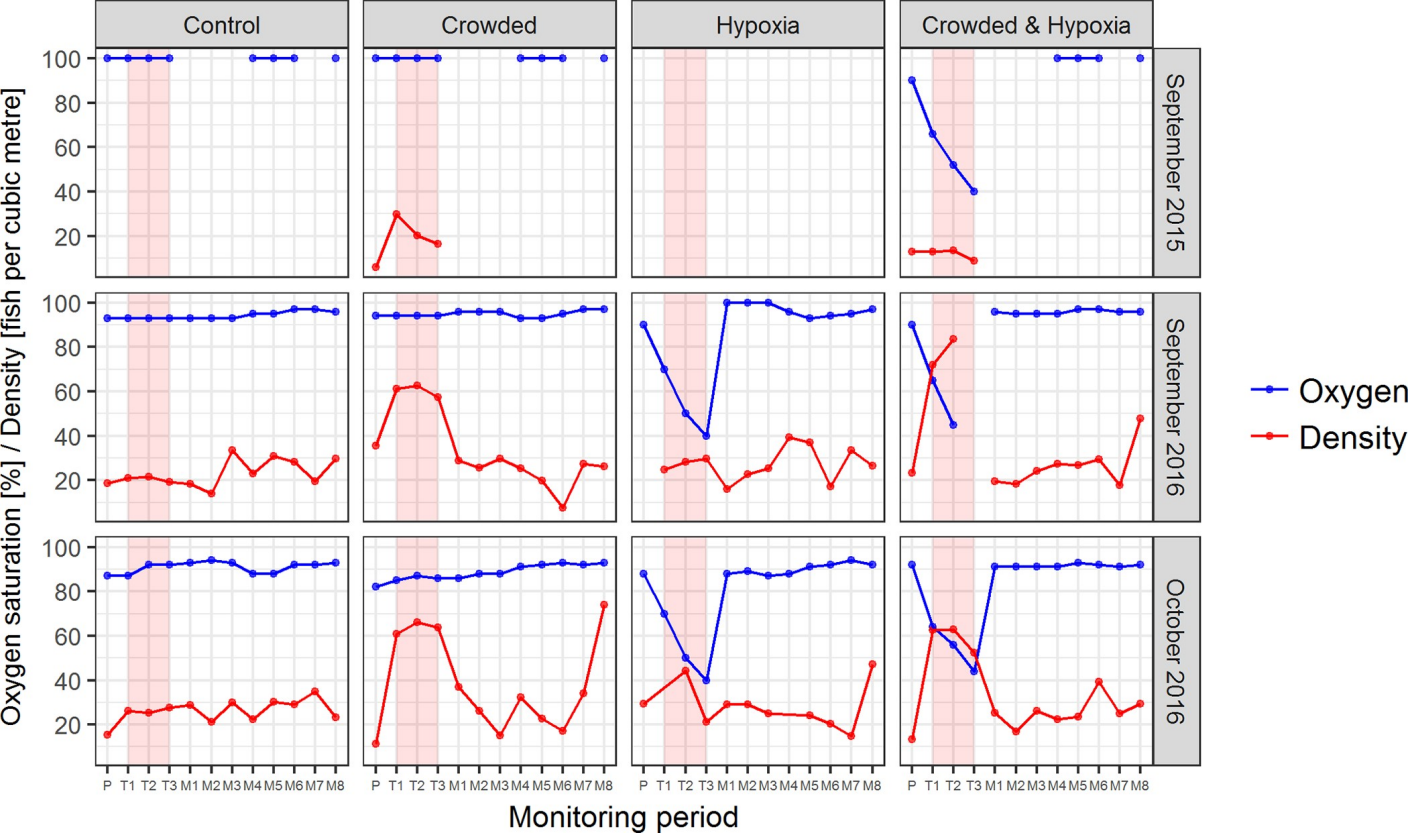
Crowding and hypoxia treatments. Oxygen (dissolved oxygen saturation, blue line) and crowding (fish density, red line) conditions throughout monitoring periods during different stressor treatments, for the three experimental phases. The red shaded area indicates the monitoring periods corresponding to the application of the stressor. For the September 2015 phase, no hypoxia treatment was applied and no density estimates were collected in the post-treatment monitoring periods or at any point during the control treatment.

During stressor treatments involving hypoxia, oxygen saturation levels dropped to the pre-determined safe threshold level of 40% (Fig 2), over a period of approximately 3 hours and rapidly returned to non-limiting levels within 10 mins or less once the tarpaulin bag was lowered. Rates of oxygen decline during treatment ranged from 11.4 to 39.0%/hour and tended to be higher in the “crowded and hypoxia” treatments (Table 2). Oxygen saturation remained constant during the “control” and “crowded” treatments (Fig 2). During treatments involving crowding, density increased by an average (mean) of 3 times compared to pre-treatment levels and rapidly returned to baseline levels post-treatment (Fig 2). Changes in density during the control and hypoxia treatments were negligible (mean: 1.2 times increase).

### Behavioural responses

The TBF data consisted of a total of 1770 records and showed variability across the monitoring periods, with clear increases in TBF compared to pre- and post-treatment levels that coincided with the application of the stressors. These increases occurred for all treatments apart from the “control”, although the increase during “hypoxia” treatment was noticeably less distinct than for the other treatments (S1 Fig). TBF rapidly returned to baseline levels following the removal of the stressor (S1 Fig). These indications were supported by the LMM fitted to the data (S1 Table), which indicated highly significant effects of the interaction term (“Monitoring period * Crowded * Hypoxia”, LRT = 67.19, df = 34, p < 0.001) and also experimental phase (LRT = 12.99, df = 2, p = 0.002).

**Fig 3:**
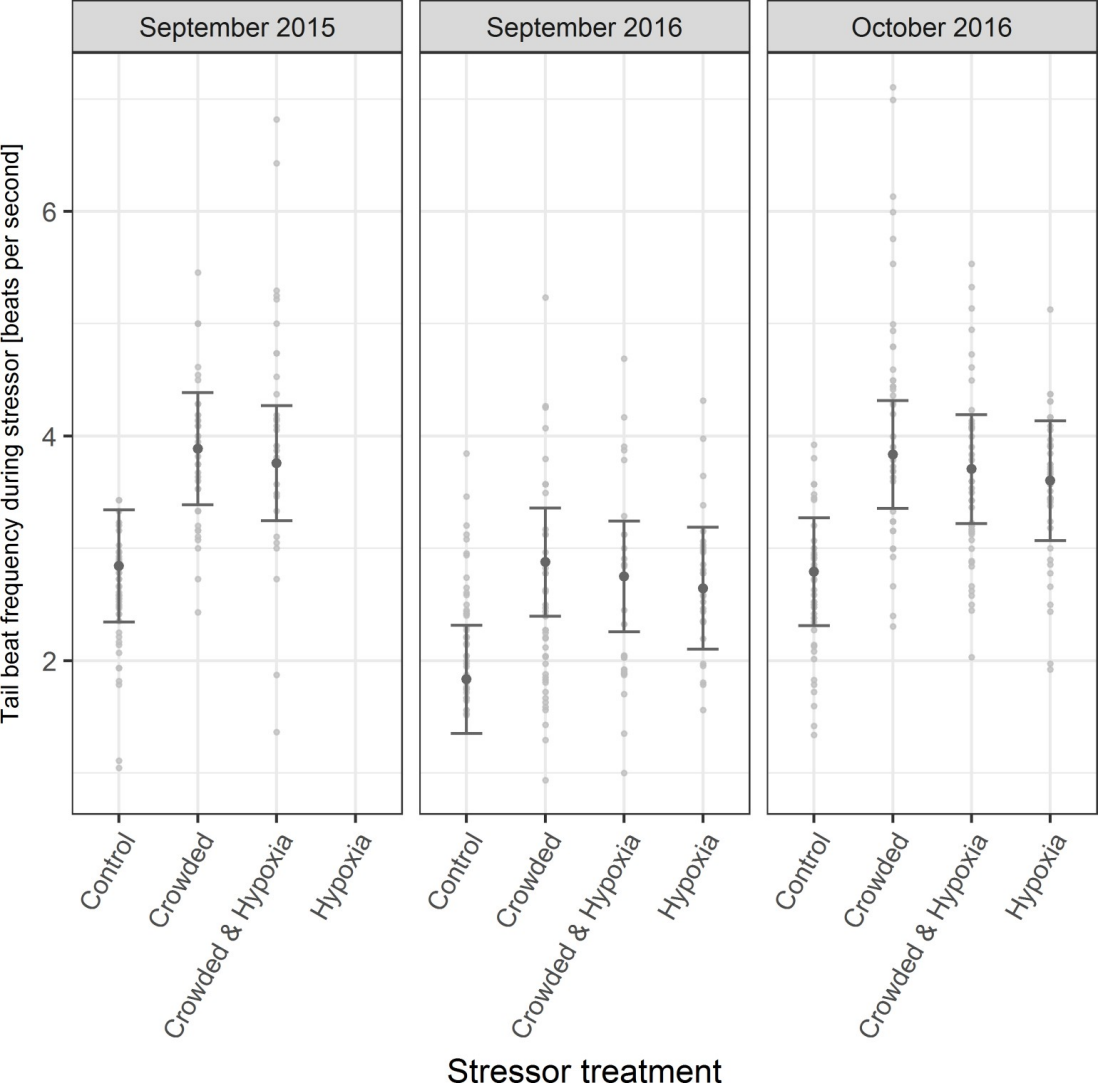
Tail beat frequency responses. Model predicted mean (± 95% confidence intervals) tail beat frequency during the application of the different stressors for the three experimental phases. No hypoxia treatment was applied during the September 2015 phase. The underlying raw data is shown as grey points.

Considering just the data from during the application of the stressor (ie. monitoring periods “T1”, “T2” and “T3”, n = 450), TBF was higher for all stressors than for their respective controls during all phases (Fig 3). The overall mean increase in TBF above “control” levels due to the “crowded”, “hypoxia” and “crowded and hypoxia” treatments was 1.04 bps (a 42% increase), 0.72 bps (a 29% increase) and 0.94 bps (a 38% increase) respectively. Differences between “crowded”, “hypoxia” and “crowded and hypoxia” TBFs within individual experimental phases were minimal (Fig 3). There were however clear differences between phases, with TBF in “September 2016” being notably lower for all treatments. The LMM fit to the data (Table 3) indicated highly significant effects of the interaction between “crowded” and “hypoxia” (LRT = 7.29, df = 3, p = 0.007), of “crowded” (LRT = 7.35, df = 1, p = 0.007) and of experimental phase (LRT = 17.76, df = 2, p < 0.001). However, “hypoxia” alone was not significant (LRT = 1.72, df = 1, p = 0.19). The pseudo R^2^ values (conditional effects pseudo R^2^ = 0.54; marginal effects pseudo R^2^ = 0.37) for the model indicated that TBF was relatively similar between different video clips and that the majority of the explained variance in the data was attributable to TBF differences between treatments and phases.

**Table 3:** Tail beat frequency during stressor modelling results. Linear mixed model coefficients for the relationship between stressor treatments (“Crowded”, “Hypoxia” and their interaction), experimental phase (“Phase”) and tail beat frequency during the application of the stressor.

Parameter	Coefficient value	S.E	Df	t-value	p-value
(Intercept)	2.84	0.26	360	11.15	<0.001
Crowded	1.04	0.28	5	3.75	0.013
Hypoxia	0.81	0.33	5	2.49	0.055
Phase: September 2016	-1.01	0.27	5	-3.69	0.014
Phase: October 2016	-0.05	0.27	5	-0.19	0.856
Crowded & Hypoxia	-0.94	0.43	5	-2.18	0.081

Preliminary sampling found no indication of a response in TBA to the stressors (Panel D of Figure A in S1 Results). Furthermore, we found no indication of a response in nearest neighbour distances (Panel A of Figure A in S1 Results) or angular deviations during the “hypoxia” or “control” treatments (Panels B and C of Figure A in S1 Results), while fish density and extreme close proximity to the stereo-camera during “crowding” and “crowding and hypoxia” prevented sampling for these treatments. Further detail of these results and methodological limitations is included in the supporting information (S1 Results).

## Discussion

In order to develop ways of monitoring welfare in commercial purse seine fishing, this study examined several behavioural metrics in individual mackerel in response to sub-lethal levels of capture stress. For the hypoxia treatment, no response in nearest neighbour distance (NND), angular deviation in pitch (ADP) or angular deviation in yaw (ADY) could be detected and likewise we detected no response in tail beat amplitude (TBA) to either “crowding” or “crowding and hypoxia” treatments. However, tail beat frequency (TBF) showed a significant increase in response to the “crowding” and “crowding and hypoxia” treatments; “hypoxia” alone showed an increased but non-significant effect. Previous studies regarding the reaction of mackerel to stress have examined physiological stress responses [80,81], while others have focused solely on mortality outcomes [22,23]. The results presented here are the first to detail individual-level behavioural responses to stress in relatively large groups of mackerel.

There was an obvious increase in TBF during the “crowded” and “crowded and hypoxia” treatments that coincided with the application of the stressors. In concurrent observations of the same fish schools, Handegard *et al*. [31] were unable to determine swimming speed for the crowded treatments using sonar imaging and optical flow algorithms due to the high densities. By using camera observations underneath the school to observe TBF, we were able to overcome this limitation. Swimming speed in fish is dependent on TBF and TBA [57] and TBF correlates positively with swimming speed in mackerel [82]. Together with our finding that TBA did not change in response to the stressors, it is reasonable to assume that the mackerel in this study increased their swimming speed in response to the treatments.

There was no indication of a behavioural response in TBA in the investigated stressors. This is likely because TBA is stable in mackerel over a wide range of swimming speeds [83]. The similarity between mean TBA observed in our study (~0.11 body lengths [BL]) and [83] supports this. Changes in TBA is more associated with acceleration rather than actual speed [57], and the range of TBA observed in the present study was very low (between 0.10–0.14). This suggests that our mackerel were swimming at steady speeds, without noteworthy acceleration or deceleration. Steady swimming speeds may, however, be the exception rather than the rule in the highly dynamic purse seine capture situation, where fish are forced to respond to a myriad of variable stressors. Such a situation is certainly different to our mesocosm net pen experiment, where fish were forced to swim in regular circular patterns. TBA cannot therefore be ruled out as a potentially useful indicator of welfare, but to determine this would require substantial alterations to our current experimental design.

The likely functional explanation for the increase in TBF is, at least for a natural situation, an adaptive response to a perceived threatening situation. Under stress, schooling fish alter their swimming speed [28] and increases in speed is a common response in pelagic schooling species [41,72,84–86]. Increases in speed allow enhanced avoidance, moving the animal away from sub-optimal areas and reducing the physiological impact of the stressor [17]. However, it is important to note that although the TBF response may be adaptive in a natural situation, this may not be the case when responding to prolonged and unavoidable anthropogenic sources of stress, such as purse seine capture. In these situations, increased activity may contribute to further hypoxia, exhaustion and increased interaction with the gear (leading to injury and scale loss), which would serve to increase post slipping mortality [86,87].

There were notable differences in absolute TBF values between different experimental phases, which was supported by the pseudo R^2^ results. These differences likely reflect differences in uncontrolled ambient conditions between phases such as availability of natural food items in the pens [88], noise levels [89], current conditions [90], temperature [91] and lighting conditions [48], all which could determine the behavioural activity level of mackerel. Likewise, differences between phases in the degree of school polarization and/or nearest neighbour distances has the potential to further explain this finding [38,92]. Furthermore, swimming speed in mackerel schools in the wild has been shown to vary with time [93,94]. Therefore, as speed/TBF naturally varies between schools and across time, it is important to consider TBF in comparison to baseline levels and therefore as a relative behavioural stress indicator. Consequently, future attempts to quantify this metric in the fishery should focus on monitoring TBF change rather than examining absolute values.

Hypoxia alone was not a significant predictor of TBF and when considering TBF across all monitoring periods (S1 Fig), the during treatment increase was less evident than for the crowding treatments. An increase in sample size may have helped to overcome this, as we conducted only 2 (rather than 3) replicates of the hypoxia treatment, experienced missing data due to camera failures (including during treatment) and observed a fair degree of variation in TBF outside of the treatment period. It is notable however that mean TBF during hypoxia treatment was 29% higher than in the controls and there was little difference in TBF during treatment between the crowding experiments and the hypoxia experiments. It would therefore seem that there is a behavioural signal in response to hypoxia stress in mackerel, but that this signal was somewhat weaker than for crowding stress.

Our hypoxia results are consistent with concurrent observations we made of swimming speed using sonar imaging (reported in [31]), where we found some indication of an increasing trend during decreasing hypoxia but no statistical significance. However, methodological limitations prevented swimming speed measurement for crowding treatments in [31]. By using camera observation we were able to demonstrate a clear TBF response not only to crowding but to the interactive effect of crowding and hypoxia as well. The presence of the dual stressors did not have a simple cumulative effect on swimming speed, as hypoxia modified the crowding response by inducing a slight reduction in TBF. Such antagonistic effects of multiple stressors may reflect a compensatory response to conserve energy when stressed in a low oxygen environment or conversely, a lack of capacity to respond fully due to the effects of hypoxia [95]. The rate of recovery to baseline levels also seemed slower for the “crowded and hypoxia” case (S1 Fig), perhaps indicating a longer lasting stress response. Either way, as oxygen in commercial purse seine operations has been observed to reduce rapidly (M. Breen, *pers*. *comm*.), future work should focus on investigating behavioural responses in mackerel to acute oxygen saturation change which may modify the response we observed.

Alternations to swimming speed have been observed in herring (*Clupea harengus*) when oxygen saturation decreases below ~35% saturation [39,42]. Prior to this level, swimming speed is stable. In the present study, we detected increases in mackerel swimming activity well above 35% saturation. Mackerel are a highly active, fast swimming species [82,88] with a relatively large proportion of red muscle [96] and are therefore highly oxyphilic; more so than herring [54], which are known to tolerate prolonged periods of hypoxia whilst overwintering [97]. This higher sensitivity to oxygen reduction may go some way to explain why we observed TBF changes in mackerel at relatively high levels of oxygen saturation.

To increase oxygen availability and decrease usage, fish in hypoxic conditions typically alter their position within the school less often [39] and increase their spacing [98]. This is contrary to our findings of no NND or angular deviation response during the hypoxia treatments. Notably, previous work has shown that some behavioural responses to hypoxia may only manifest below a critical, near-lethal, level and depend on the rate of oxygen change [39,55]. Our declines in oxygen took place over a period of ~3 hours (the fastest being ~1.5 hours, [31]) and purposely did not fall below ~40% saturation, suggesting that the stressor we applied was not acute or rapid enough to elicit a measurable response for these metrics.

During treatments involving crowding, the lack of available swimming space presumably forced the fish towards the net pen wall and therefore the stereo-camera. With our system, fish were required to be at least 0.6m away from the lenses to allow stereo-photogrammetry, meaning we were unable to quantify any NND or angular deviation responses to the “crowding and “crowding and hypoxia” treatments. This issue could be overcome by either reducing the separation between the two lenses or by increasing the distance between the camera and the measured fish, but both solutions would negatively affect the precision of the stereo-photogrammetric measurements.

The mean NND in our study (~0.7 BL) corresponds well to previous observations of mackerel spacing (0.3–0.9 BL) [48,49]. However, our mean ADY (2°) is substantially lower than previous estimates (6–14°) [48,49] for circling mackerel. This difference is likely attributable to the much smaller school sizes (up to 110 fish) examined by [48,49] compared to our study (~1600 fish). As a result, fish likely swam in tighter circles thereby increasing the angular deviation between nearest neighbours relative to our study.

Our experimental design ensured that fish were only exposed to stressor treatments once, minimising the possibility of habituation to repeated stimuli [99]. However, prior to experimentation the fish had been held in the holding net pen for up to a year and in the experimental nets for a minimum of 7 days, which would have almost certainly allowed some habituation to the captive environment. Consequently, a reduction in the behavioural response to our stressors can be expected in comparison to truly wild fish. This is of particular relevance because our crowding stressor treatments were induced by manipulating the volume of the net pen itself. Despite this, we still observed a clear behavioural response in TBF suggesting that this is a robust indictor of sublethal stress. This is further supported by the fact that the TBF signal was seen throughout the different crowding treatments, despite differences in enforced density (which arose due to differences in fish abundance between pens), as well as the lack of response in the control experiments. It is now important to determine if and how this indicator is modified when observing wild fish during the capture process.

Although efforts are underway to reduce the need for slipping by improving pre catch characterisation using new technologies [100], slipping still occurs during mackerel purse seining and is a legal but regulated practice in Norwegian [101] and EU [102,103] waters. Current legislation recognises the particular vulnerability of mackerel during the final stage of capture [23] and indicates that exposure to fatal crowding levels must be avoided by releasing fish before a certain proportion of the net is hauled. Our findings of an indicator of sub-lethal stress in mackerel has the potential to help to avoid such fatal exposures via the monitoring of fish behaviour. Extending the relationship between capture stress and the TBF response to include mortality outcomes would be informative for further reducing post slipping mortality via behavioural observation. For this, the variability in degree and duration of stress typically experienced by the fish during the final stages of capture should be accurately characterised using observations from the fishery.

In conclusion, the results of this study have highlighted a clear behavioural indicator of sublethal capture related stressors, namely an increase in TBF. To be able to utilise this signal as a measure of welfare during purse seine capture, it must first be established that mackerel display the same response in the field. From there, the challenge will then be to develop methods to be able to reliably observe this metric so that real time decisions regarding welfare can be made. It is doubtful that the method employed in the present study to determine TBF (i.e. camera observation and subsequent manual counting) has utility in a real commercial catch situation, although deploying camera systems into the purse seine may allow confirmation that the TBF signal exists in commercial fisheries. More practical however is the use of split beam echosounders, which have been used to successfully quantify TBF remotely in dense schools of herring [104]. Such techniques may have real utility for monitoring of mackerel welfare, in order to minimise capture related stress and maximise post slipping survival. However, this will depend upon the technique be successfully adapted to this species and to the often challenging acoustic environment inside of the purse seine net [105,106].

## Supporting information

S1 Methods Stereocamera system setup. Detail of the stereo-camera system, calibration and deployment strategy.
(DOCX)

S2 Methods Measurement of the tail beat amplitude, nearest neighbour distance and nearest neighbour angular deviation behavioural metrics. Detail of how cartesian coordinates were used to determine nearest neighbour distances and angular deviations and how tail beat amplitude was quantified.
(DOCX)

S3 Methods Statistical analysis procedures. Statistical analysis details for the tail beat amplitude, nearest neighbour distance and nearest neighbour angular deviation behavioural metrics.
(DOCX)

S1 Fig Tail beat frequency responses throughout all monitoring periods. Model predicted mean (± 95% confidence intervals) tail beat frequency across all monitoring periods for different stressor treatments in the three experimental phases. The red shaded area indicates the monitoring periods corresponding to the application of the stressor. The underlying raw data is shown as grey points. No hypoxia treatment was applied during the September 2015 phase. The “T3” monitoring period for crowded and hypoxia treatment in September 2016 was not collected due to rapidly falling oxygen saturation in the net pen. In the hypoxia treatment, camera failures account for the missing data for “T2” in in September 2016 and “P” in October 2016.
(TIF)

S1 Table Tail beat frequency modelling results for all monitoring periods. Linear mixed model coefficients for the relationship between tail beat frequency and monitoring periods (included in the model as “Monitoring code”), the stressors treatments (“Crowded”, “Hypoxia” and their interaction) and experimental phase (“Phase”).
(DOCX)

S1 Results Tail beat amplitude, nearest neighbour distance and nearest neighbour angular deviation results. Detail and visualisation of tail beat amplitude, nearest neighbour distance and nearest neighbour angular deviation results, including description of methodological issues encountered during stereo-camera observation of behaviour during “crowding” and “crowding and hypoxia” treatments.
(DOCX)

S1 Dataset Tail beat frequency (TBF) dataset. (CSV)

S2 Dataset Tail beat amplitude (TBA) dataset. (CSV)

S3 Dataset Nearest neighbour distance (NND) dataset. (CSV)

S4 Dataset Nearest neighbour angular deviation in pitch (ADP) dataset. (CSV)

S5 Dataset Nearest neighbour angular deviation in yaw (ADY) dataset. (CSV)

S1 Video Randomly selected examples of the perspective given by the vertically orientated GoPro camera. Containing examples of both crowding and non-crowding treatments.
(MP4)
