# Supplementary material for: Effects on individual level behaviour in mackerel (Scomber scombrus) of sub-lethal capture related stressors: Crowding and hypoxia
Source: PLoS One. 2019 Mar 13;14(3):e0213709. doi: 10.1371/journal.pone.0213709 (PMC6415853; doi:10.1371/journal.pone.0213709)
Supplement: S1 Methods — Detail of the stereo-camera system, calibration and deployment strategy. (DOCX) [file pone.0213709.s001.docx]

**S1 Methods**

**Stereo-camera system setup**

The stereo-camera consisted of two identical colour Luxus Compact Cameras (MacArtney Underwater Technology) filming at 25fps. These were securely fixed in a metal frame positioned 0.6 m apart and at an offset angle of 6° towards each other. Synchronisation of video frames between the two cameras was ensured by simultaneously filming a flashing reference light on both cameras, at the start and end of each observation period.

The stereo-camera was placed against the pen wall (Figure 1 in main text), at varying depths depending on the vertical positioning of the school in the pen. Each monitoring period lasted 10 minutes, consisting of two consecutive observation stages of 5 minutes each. For the first stage (St-1), the stereo-camera recorded the school frontally, with the intention of filming the positioning of individual fish relative to the school as they swam approximately perpendicular to the camera. For the second stage (St-2), the stereo-camera was turned slightly left or right around its vertical axis, to record the fish swimming obliquely to the camera.

Prior to each experimental phase, the optical properties, positioning and orientation of the two cameras in the stereo-camera system was calibrated by collecting footage of a “calibration cube” of known dimensions in conjunction with SeaGIS CAL software (<http://www.seagis.com.au>). The software performed a bundle adjustment to produce a cartesian coordinate system for images produced by the cameras. Using SeaGIS EventMeasure software, the coordinate system can then be used to make stereo-photogrammetric measurements from paired synchronised images.

Within each monitoring period, we randomly selected 6 paired images of sufficient lighting quality for analysis; 3 images from St-1 and 3 from St-2. From each paired image, we attempted to collect as many behavioural metrics as possible from all visible fish within the image.
