# Supplementary material for: Effects on individual level behaviour in mackerel (Scomber scombrus) of sub-lethal capture related stressors: Crowding and hypoxia
Source: PLoS One. 2019 Mar 13;14(3):e0213709. doi: 10.1371/journal.pone.0213709 (PMC6415853; doi:10.1371/journal.pone.0213709)
Supplement: S2 Methods — Detail of how cartesian coordinates were used to determine nearest neighbour distances and angular deviations and how tail beat amplitude was quantified. (DOCX) [file pone.0213709.s002.docx]

**S2 Methods**

**Measurement of the tail beat amplitude, nearest neighbour distance and nearest neighbour angular deviation behavioural metrics**

Mean tail beat amplitude as a proportion of total body length (TBA) was collected from the same fish as tail beat frequency, by measuring the distance in pixels from the midline of the mackerel body to the extreme lateral position of a tail beat using Image J. This distance was collected from all tail beats during the fish’s appearance on camera, then converted to a proportion of the total body length of the fish (also measured in pixels). Proportions were then averaged to give a mean TBA for the time the fish was in the field of view.

When discussing orientation angles throughout this work, pitch ($P$) refers to rotation around the lateral (left-right) axis of the fish; yaw ($Y$) refers to rotation around the dorsoventral axis (that is, the axis perpendicular to the laterally formed axis) and roll ($R$) refers to rotation around the anteroposterior axis. As schooling position and angle is chiefly mediated by the visual sense (Partridge & Pitcher, 1980), nearest neighbour distance (NND) was defined as the distance between the eye of the target fish and the eye of its nearest neighbour. However, the collected footage contained considerable amounts of occultation as the fish schooled tightly and passed between others and the camera. This meant that often we could see parts of fish within the images but could not determine their eye position, potentially greatly reducing the number of fish from which behavioural metrics could be gathered. We therefore used morphometrical measurements of other fish to predict the eye position of fish which were visible to some degree but who’s eye position was occulted. To allow predictions, we determined the position of the following morphometric features when possible: tip of the snout (referred to as the “head”), the posterior end of the middle caudal fin ray (the “tail”), the posterior end of the caudal penduncle (the “penducle”), the proximal end of the pectoral fin (the “pectoral”) and the eye position on as many fish as possible within all analysed images. These positions were then used to calculate median distances (D) between the eye position and the other morphometric features. Accordingly, in cases were the eye position of a fish was not visible we predicted its cartesian position depending on the typical distance between a visible morphometric feature and the eye as well as either: 1) the pitch, roll and yaw of the target (but eye position occulated) fish, or for cases of strong occlusion where it was not possible to calculate angles of the target fish: 2) the mean pitch, roll and yaw of other fish within the image. For this, the following equations were used: $x_{eye}=x_{1}-D \cdot\cos R$, $y_{eye}=y_{1}-D\cdot\cos P$ and $z_{eye}=z_{1}-D\cdot\cos Y$, where $x_{1}$,$y_{1}$,$z_{1}$ are the cartesian positions of the used morphometric feature, $D$ the median distance between the used morphometric feature and the eye and $R$,$P$,$Y$ the roll, pitch and yaw, respectively, of the target fish, or the mean roll, pitch and yaw respectively of other fish within in the image, if required.

Having established the position of eyes, NND in millimetres was calculated using Euclidean distances. Roll ($R$), pitch ($P$) and yaw ($Y$) were determined by calculating the angle in radians between the most distally placed morphometric features visible on the target fish (for instance, head and tail orientation would be preferred over head to pectoral orientation) using the following equations:

$R=\arccos\left( \frac{\Delta y}{\sqrt{{\Delta x}^{2}+{\Delta y}^{2}+{\Delta z}^{2}}} \right)$,

$P=\arccos\left( \frac{\Delta x}{\sqrt{{\Delta x}^{2}+{\Delta y}^{2}+{\Delta z}^{2}}} \right)$ and

$Y=\arccos\left( \frac{\Delta z}{\sqrt{{\Delta x}^{2}+{\Delta y}^{2}+{\Delta z}^{2}}} \right)$,

where $\Delta y$,$\Delta x$ and $\Delta z$ are the differences between the two cartesian positions of the used morphometric features.

Where insufficient morphometric features were available due to occlusion to calculate angles, the mean pitch, roll and yaw of other fish within the image was applied. Consequently, angular deviation in pitch (ADP) was calculated using

$ADP=P_{2}-P_{1}$,

where $P_{1}$ is the pitch in radians of the target fish and $P_{2}$ is the pitch in radians of the target fish’s nearest neighbour.

Angular deviation in yaw (ADY) was calculated as

$ADY=Y_{2}-Y_{1}$,

where $Y_{1}$ is the yaw in radians of the target fish and $Y_{2}$ is the yaw in radians of the target fish’s nearest neighbour.

It was obviously not possible to determine the eye position and angles of fish which were completely occulted, or not included in the field of view of both cameras. Therefore, our behavioural measures of nearest neighbour distance and nearest neighbour angular deviation are perhaps more correctly referred to as nearest *visible* neighbour distance and angular deviation, because we could not guarantee that the actual nearest neighbour was included in the sampling.

**References**

Partridge, B. L., & Pitcher, T. J. (1980). The sensory basis of fish schools: relative roles of lateral line and vision. *Journal of Comparative Physiology*, *135*(4), 315-325.
