# Supplementary material for: Effects on individual level behaviour in mackerel (Scomber scombrus) of sub-lethal capture related stressors: Crowding and hypoxia
Source: PLoS One. 2019 Mar 13;14(3):e0213709. doi: 10.1371/journal.pone.0213709 (PMC6415853; doi:10.1371/journal.pone.0213709)
Supplement: S3 Methods — Statistical analysis details for the tail beat amplitude, nearest neighbour distance and nearest neighbour angular deviation behavioural metrics. (DOCX) [file pone.0213709.s003.docx]

**S3 Methods**

**Statistical analysis procedures for the nearest neighbour distance, nearest neighbour angular deviation and tail beat amplitude behavioural metrics**

We modelled nearest neighbour distances (NND), angular deviation in pitch (ADP), angular deviation in yaw (ADY) and tail beat amplitude (TBA) using mixed models. A correlation structure of “monitoring period” nested within “video footage” (either the individual paired frame from the stereo-camera or the five second clip from the GoPro footage) was initially included as random effects. In some cases, the complexity of the attempted correlation structure meant that some models would not converge. In such cases, we included “video footage” only as a random effect.

For the NND, ADP and ADY models, we included a fixed effect of an interaction between “monitoring period” and “stressor treatment” (two levels, either hypoxia or control). For the TBA model, we included fixed effects of “crowded”, and “hypoxia” (dummy variables coded as 1 for presence and 0 for absence) as separate effects along with monitoring code. Missing data prevented the inclusion of an interaction term for the TBA model.

The response variables of ADP and ADY represented continuous data, but with a theoretical lower bound of zero because angular deviations were expressed as absolute differences. However, ADP and ADY values were always >0 and the data was highly positively skewed. We therefore modelled these behavioural variables using a gamma distribution and log link (Zuur *et al.,* 2009) via generalised linear mixed modelling (GLMM). We used the glmer function of the lme4 library of R (Bates *et al.,* 2015), with the following R syntax:

(1) glmer(ADP ~ Monitoring_period * Stressor_treatment +

(1 | Monitoring_period/Video_footage),

family = Gamma(link = "log"))

(2) glmer(ADY ~ Monitoring_period * Stressor_treatment +

(1 | Monitoring_period/Video_footage),

family = Gamma(link = "log"))

The NND response variable represented normally (continuous) distributed data and was modelled using linear mixed models (LMM). Although the response variable TBA was binomially distributed (representing proportions of total body length), validation plots of model residuals indicated that it could also be modelled successfully using LMM. Consequently, the R syntax for the final models was as follows:

(3) lme(NND ~ Monitoring_period * Stressor_treatment,

random = ~ 1 | Video_footage,

weights = varIdent(form = ~1 | Monitoring_code),

method = "REML")

(4) lme(TBA ~ Monitoring_code + Crowded + Hypoxia,

random = ~ 1 | Video_footage,

weights = varIdent(form = ~1 | Monitoring_code),

method = "REML")

**References**

Bates, D., Maechler, M., Bolker, B., & Walker, S. (2015). Fitting Linear Mixed-Effects Models Using lme4. Journal of Statistical Software, 67(1), 1-48.

Zuur AF, Ieno EN, Walker NJ, Savaliev AA, Smith GM (2009) Mixed effects models and extensions in R. Gail M, Krickeberg K, Samet JM, Tsiatis A, Wong W, editors. New York: Springer.
