# Supplementary figures and images for: Effects on individual level behaviour in mackerel (Scomber scombrus) of sub-lethal capture related stressors: Crowding and hypoxia
Source: PLoS One. 2019 Mar 13;14(3):e0213709. doi: 10.1371/journal.pone.0213709 (PMC6415853; doi:10.1371/journal.pone.0213709)

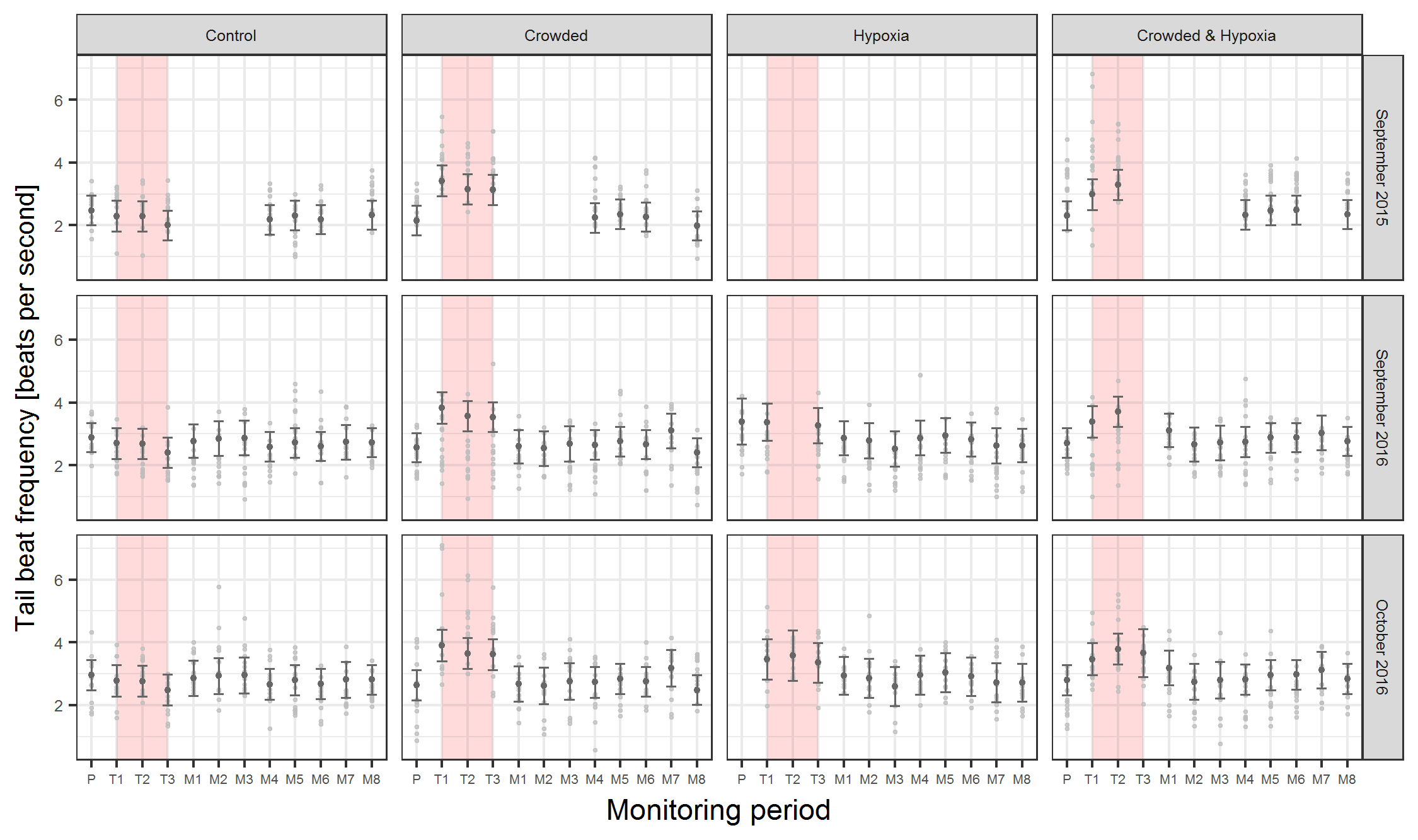

Supplement: S1 Fig — Model predicted mean (± 95% confidence intervals) tail beat frequency across all monitoring periods for different stressor treatments in the three experimental phases. The red shaded area indicates the monitoring periods corresponding to the application of the stressor. The underlying raw data is shown as grey points. No hypoxia treatment was applied during the September 2015 phase. The “T3” monitoring period for crowded and hypoxia treatment in September 2016 was not collected due to rapidly falling oxygen saturation in the net pen. In the hypoxia treatment, camera failures account for the missing data for “T2” in in September 2016 and “P” in October 2016. (TIF) [file pone.0213709.s004.tif]
