# Supplementary material for: Effects on individual level behaviour in mackerel (Scomber scombrus) of sub-lethal capture related stressors: Crowding and hypoxia
Source: PLoS One. 2019 Mar 13;14(3):e0213709. doi: 10.1371/journal.pone.0213709 (PMC6415853; doi:10.1371/journal.pone.0213709)
Supplement: S1 Table — Linear mixed model coefficients for the relationship between tail beat frequency and monitoring periods (included in the model as “Monitoring code”), the stressors treatments (“Crowded”, “Hypoxia” and their interaction) and experimental phase (“Phase”). (DOCX) [file pone.0213709.s005.docx]

| **Parameter** | **Coefficient value** | **S.E** | **df** | **t-value** | **p-value** |
| --- | --- | --- | --- | --- | --- |
| (Intercept) | 2.857 | 0.285 | 1392 | 10.041 | 0.000 |
| Monitoring_codeM2 | 0.079 | 0.334 | 61 | 0.238 | 0.813 |
| Monitoring_codeM3 | 0.096 | 0.336 | 61 | 0.287 | 0.775 |
| Monitoring_codeM4 | -0.188 | 0.306 | 61 | -0.614 | 0.541 |
| Monitoring_codeM5 | -0.057 | 0.305 | 61 | -0.187 | 0.852 |
| Monitoring_codeM6 | -0.175 | 0.302 | 61 | -0.581 | 0.563 |
| Monitoring_codeM7 | -0.039 | 0.334 | 61 | -0.118 | 0.907 |
| Monitoring_codeM8 | -0.042 | 0.301 | 61 | -0.140 | 0.889 |
| Monitoring_codeP | 0.108 | 0.303 | 61 | 0.356 | 0.723 |
| Monitoring_codeT1 | -0.075 | 0.315 | 61 | -0.238 | 0.813 |
| Monitoring_codeT2 | -0.088 | 0.309 | 61 | -0.285 | 0.777 |
| Monitoring_codeT3 | -0.369 | 0.307 | 61 | -1.203 | 0.234 |
| Crowding | -0.172 | 0.364 | 5 | -0.473 | 0.656 |
| Hypoxia | 0.082 | 0.372 | 5 | 0.221 | 0.834 |
| PhaseSeptember 2016 | -0.486 | 0.175 | 5 | -2.781 | 0.039 |
| PhaseOctober 2016 | -0.081 | 0.175 | 5 | -0.462 | 0.663 |
| Monitoring_codeM2:Crowding | -0.143 | 0.472 | 61 | -0.303 | 0.763 |
| Monitoring_codeM3:Crowding | -0.015 | 0.475 | 61 | -0.031 | 0.975 |
| Monitoring_codeM4:Crowding | 0.236 | 0.432 | 61 | 0.545 | 0.588 |
| Monitoring_codeM5:Crowding | 0.216 | 0.431 | 61 | 0.502 | 0.617 |
| Monitoring_codeM6:Crowding | 0.244 | 0.426 | 61 | 0.572 | 0.569 |
| Monitoring_codeM7:Crowding | 0.536 | 0.472 | 61 | 1.137 | 0.260 |
| Monitoring_codeM8:Crowding | -0.159 | 0.425 | 61 | -0.373 | 0.710 |
| Monitoring_codeP:Crowding | -0.149 | 0.428 | 61 | -0.348 | 0.729 |
| Monitoring_codeT1:Crowding | 1.298 | 0.445 | 61 | 2.917 | 0.005 |
| Monitoring_codeT2:Crowding | 1.048 | 0.436 | 61 | 2.405 | 0.019 |
| Monitoring_codeT3:Crowding | 1.303 | 0.433 | 61 | 3.006 | 0.004 |
| Monitoring_codeM2:Hypoxia | -0.152 | 0.472 | 61 | -0.322 | 0.749 |
| Monitoring_codeM3:Hypoxia | -0.431 | 0.475 | 61 | -0.908 | 0.367 |
| Monitoring_codeM4:Hypoxia | 0.203 | 0.452 | 61 | 0.449 | 0.655 |
| Monitoring_codeM5:Hypoxia | 0.155 | 0.451 | 61 | 0.343 | 0.733 |
| Monitoring_codeM6:Hypoxia | 0.151 | 0.445 | 61 | 0.338 | 0.736 |
| Monitoring_codeM7:Hypoxia | -0.188 | 0.472 | 61 | -0.399 | 0.691 |
| Monitoring_codeM8:Hypoxia | -0.181 | 0.444 | 61 | -0.407 | 0.686 |
| Monitoring_codeP:Hypoxia | 0.427 | 0.509 | 61 | 0.838 | 0.405 |
| Monitoring_codeT1:Hypoxia | 0.593 | 0.467 | 61 | 1.268 | 0.210 |
| Monitoring_codeT2:Hypoxia | 0.737 | 0.523 | 61 | 1.409 | 0.164 |
| Monitoring_codeT3:Hypoxia | 0.783 | 0.454 | 61 | 1.726 | 0.090 |
| Crowding:Hypoxia | 0.423 | 0.521 | 5 | 0.812 | 0.454 |
| Monitoring_codeM2:Crowding:Hypoxia | -0.227 | 0.668 | 61 | -0.340 | 0.735 |
| Monitoring_codeM3:Crowding:Hypoxia | -0.038 | 0.672 | 61 | -0.056 | 0.955 |
| Monitoring_codeM4:Crowding:Hypoxia | -0.614 | 0.625 | 61 | -0.981 | 0.330 |
| Monitoring_codeM5:Crowding:Hypoxia | -0.540 | 0.623 | 61 | -0.866 | 0.390 |
| Monitoring_codeM6:Crowding:Hypoxia | -0.437 | 0.617 | 61 | -0.709 | 0.481 |
| Monitoring_codeM7:Crowding:Hypoxia | -0.380 | 0.667 | 61 | -0.570 | 0.571 |
| Monitoring_codeM8:Crowding:Hypoxia | 0.033 | 0.615 | 61 | 0.054 | 0.957 |
| Monitoring_codeP:Crowding:Hypoxia | -0.780 | 0.665 | 61 | -1.173 | 0.245 |
| Monitoring_codeT1:Crowding:Hypoxia | -1.534 | 0.645 | 61 | -2.377 | 0.021 |
| Monitoring_codeT2:Crowding:Hypoxia | -1.102 | 0.681 | 61 | -1.618 | 0.111 |
| Monitoring_codeT3:Crowding:Hypoxia | -1.245 | 0.689 | 61 | -1.808 | 0.076 |
