# Supplementary material for: Effects on individual level behaviour in mackerel (Scomber scombrus) of sub-lethal capture related stressors: Crowding and hypoxia
Source: PLoS One. 2019 Mar 13;14(3):e0213709. doi: 10.1371/journal.pone.0213709 (PMC6415853; doi:10.1371/journal.pone.0213709)
Supplement: S1 Results — Detail and visualisation of tail beat amplitude, nearest neighbour distance and nearest neighbour angular deviation results, including description of methodological issues encountered during stereo-camera observation of behaviour during “crowding” and “crowding and hypoxia” treatments. (DOCX) [file pone.0213709.s006.docx]

**S1 Results**

**Tail beat amplitude, nearest neighbour distance and nearest neighbour angular deviation results.**

Tail beat amplitude

From the GoPro camera footage, we randomly selected the September 2015 footage from which to begin quantifying the behavioural metric of tail beat amplitude (TBA). TBA changed little during the individual “control”, “crowded” and “crowded and hypoxia” experiments (Panel D in Fig A below), ranging from 0.1 to 0.11, 0.11 to 0.14 and from 0.1 to 0.12 body lengths (BL) distance respectively. Significant effects of crowding (LRT = 32.82, df = 1, p < 0.0001), hypoxia (LRT = 19.39, df = 1, p < 0.0001) and monitoring period (LRT = 19.773, df = 7, p = 0.006) were determined by the model fitted to the TBA data. However, visual inspection of the model results (Panel D in Fig A below) showed no indication that the detected differences were attributable to differences in TBA during the application of the stressors. Sampling of TBA from the September 2016 and October 2016 phases was therefore not undertaken.

Nearest neighbour distance and nearest neighbour angular deviation

Using the stereo-camera images, we randomly selected the October 2016 phase with which to begin behavioural quantification. The video showed that fish typically schooled towards the centre of the net pen. However, during the application of the stressor for treatments involving crowding, the available swimming volume was much reduced and the fish were forced closer to the net pen walls and consequently closer to the stereo-camera. This meant that fewer fish were visible in the cameras field of view and/or fish were not contained within the field of view of both of the lenses, preventing stereo-photogrammetric measurement. As a result, nearest neighbours could not be identified due to extreme occlusion or lack of image overlap between lenses**.** Examination of all video footage indicated that the same problems occurred in all phases. We therefore could not include “crowded” and “crowded and hypoxia” treatments in our sampling and focused solely on “hypoxia” and “control” treatment for the footage collected by stereo-camera.

Mean nearest neighbour distances (NND) remained fairly constant across the monitoring periods (Panel A in Fig A below), ranging from 216 to 336 mm during the “control” experiment and between 238 and 315 mm during the “hypoxia” experiment. Similarly, changes in mean angular deviation in yaw (ADY) were minimal (Panel C in Fig A below) with differences between nearest neighbours ranging between 0.02 and 0.04 radians for the “control” experiment and between 0.02 and 0.03 radians for the “hypoxia” treatment. These observations are supported by the models fitted to the NND and ADP data, where no significant effects of the interaction between stressor treatment and monitoring period were detected (LMM for NND data, LRT = 15.31, df = 10, p = 0.12; LMM for ADY data, LRT = 4.94, df = 10, p = 0.89).

Changes in mean angular deviation in pitch (ADP) during the “control” and “hypoxia” experiments (Panel B in Fig A below) were also small, ranging from 0.02 to 0.07 radians and between 0.01 and 0.07 radians respectively. However, the model fitted to the ADP data demonstrated a significant effect of the interaction between stressor treatment and monitoring period (LRT = 27.43, df = 10, p < 0.01). Despite this, visual inspection of the model results (Panel B in Fig A below) would suggest the detected differences are likely attributable to the exceptionally low ADP during the pre-treatment monitoring period (“P”) rather than due to consistent differences arising during the application of the “hypoxia” stressor. Similar variability in ADP during the “control” experiment support the notion that ADP was a naturally variable metric.

There was therefore no indication of meaningful results for the NND, ADY or ADP metrics in response to the stressor treatments. Consequently, further sampling of the remaining stereo-camera footage from the September 2015 and September 2016 phases was not undertaken.


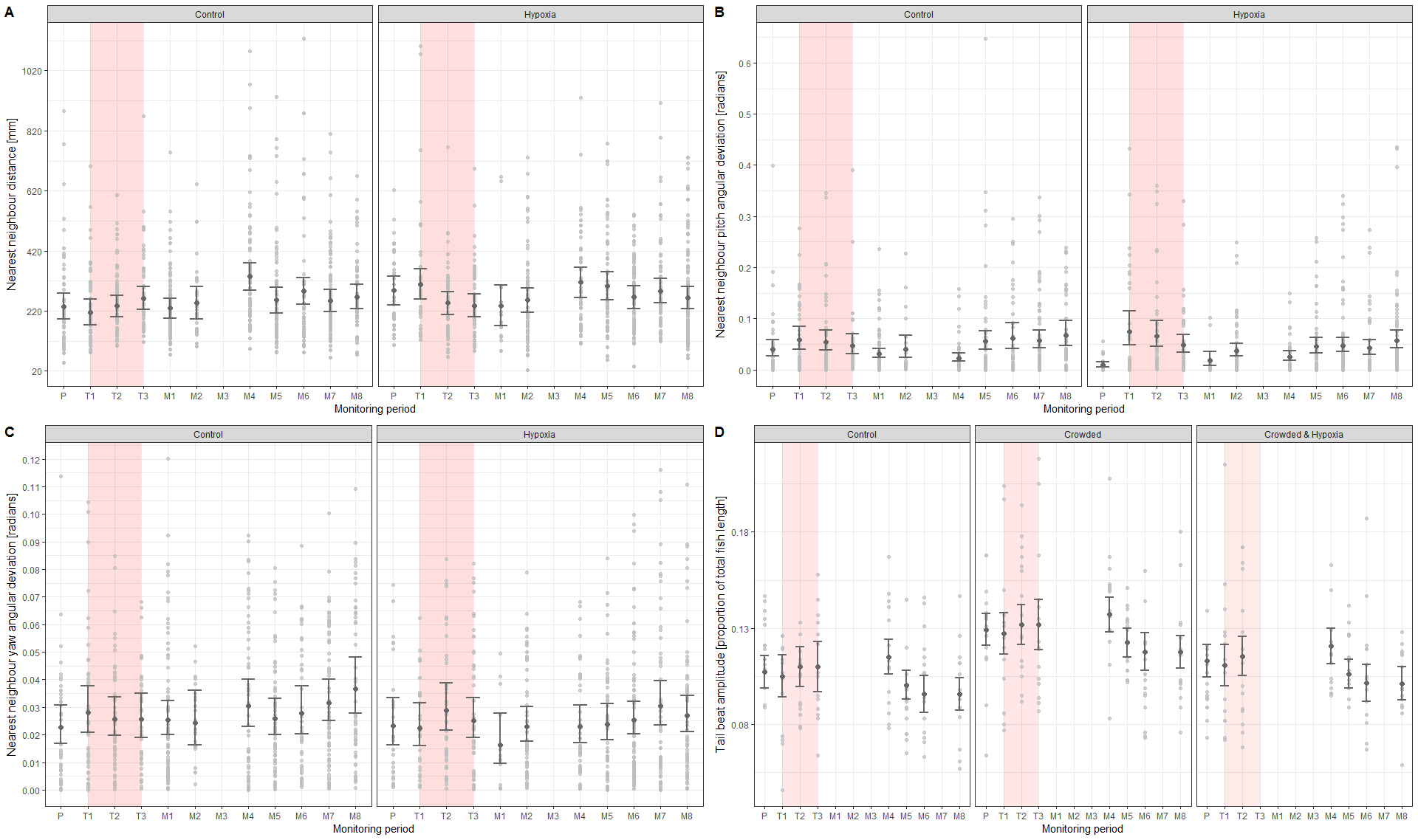


**Fig A:** Model predicted mean (± 95% confidence intervals) nearest neighbour distance (**A**), nearest neighbour angular deviation in pitch (**B**), nearest neighbour angular deviation in yaw (**C**) and tail beat amplitude (**D**) across the monitoring periods for various stressor treatments. The red shaded area indicates the monitoring periods corresponding to the application of the stressor. The underlying raw data is shown as grey points. Data for plots **A**, **B** and **C** was collected from the October 2016 phase only. Poor quality images prevented the collection of data from “M3” monitoring period. Data for plot **D** was only collected from the September 2015 phase. Camera failure at monitoring period T3 during the crowded and hypoxia stressor treatment for plot **D** prevented data collection.
